# Disparities in the Timing of Preoperative Hemodialysis Among Patients With End-Stage Kidney Disease

**DOI:** 10.1001/jamanetworkopen.2023.26326

**Published:** 2023-07-28

**Authors:** Vikram Fielding-Singh, Matthew W. Vanneman, Arden M. Morris, Glenn M. Chertow, Eugene Lin

**Affiliations:** 1 Department of Anesthesiology, Perioperative and Pain Medicine, Stanford University School of Medicine, Stanford, California; 2 Stanford Cardiovascular Institute, Stanford University School of Medicine, Stanford, California; 3 S-SPIRE Center, Stanford University, Stanford, California; 4 Department of Surgery, Stanford University School of Medicine, Stanford, California; 5 Division of Nephrology, Department of Medicine, Stanford University School of Medicine, Stanford, California; 6 Department of Internal Medicine, University of Southern California, Los Angeles; 7 Leonard D. Schaeffer Center for Health Policy & Economics, University of Southern California, Los Angeles

## Abstract

This cohort study evaluates the association of patient characteristics with the interval between hemodialysis and surgery among patients with end-stage kidney disease.

## Introduction

Patients with end-stage kidney disease (ESKD) experience significantly elevated perioperative risk compared with patients with normal or near normal kidney function.^1^ We recently observed an association between longer intervals between preoperative hemodialysis and surgery and postoperative mortality.^2^ Little is known, however, about whether minoritized or otherwise socially vulnerable patients with ESKD experience longer preoperative intervals between hemodialysis and surgery. We hypothesized that age, sex, race and ethnicity, and social deprivation are associated with variability in preoperative hemodialysis timing.

## Methods

In this cohort study, we identified adults (aged ≥18 years) with prevalent ESKD receiving hemodialysis undergoing surgical procedures from January 1, 2011, to September 30, 2018, from the United States Renal Data System (USRDS). Details of the patient cohort, dialysis treatments, and procedures have been previously described.^2^ The Stanford University institutional review board provided a waiver of participant consent according to 45 CFR §46.104. This study followed STROBE reporting guidelines.^3^

The primary exposures were age, sex, race and ethnicity, and social deprivation index.^4^ Consistent with the USRDS Annual Report,^5^ race and ethnicity was categorized as Hispanic (any race), non-Hispanic Asian, non-Hispanic Black, and non-Hispanic White. We excluded patients who identified as American Indian or Alaska Native, Pacific Islander, Middle Eastern or Arabian, Indian (subcontinent), or other or unknown owing to small population sizes and insufficient power. The primary outcome was the proportion of procedures with a 2- or 3-day interval between the last outpatient hemodialysis session and the surgical procedure. A 2- or 3-day interval is associated with higher postoperative mortality compared with a 0- or 1-day interval.^2^ We identified disparities in preoperative hemodialysis timing using a logistic regression adjusted for patient and procedural covariates and with robust variance estimation to account for patient-level clustering. Model specifications and the approach to missing data are available in the eAppendix and eTable in Supplement 1. Analyses were performed using Stata version 17.1 (StataCorp). A 2-sided Bonferroni-corrected *P* value threshold of .006 (.05 / 8 hypotheses) was used to determine statistical significance.

## Results

We identified 1 120 763 procedures among 338 391 patients. Of these, 81.9% of procedures had a shorter (0- or 1-day) interval between the last outpatient hemodialysis treatment and surgery and 18.1% had a longer (2 or 3-day) interval (Table). Compared with patients aged 18 to 39 years, older patients had longer hemodialysis-surgery intervals (age 60 to 79 years: adjusted odds ratio [aOR], 1.08 [99.4% CI, 1.02-1.15]; *P* < .001; age ≥80 years: aOR, 1.13 [99.4% CI, 1.05-1.21]; *P* < .001). Similarly, women (vs men: aOR, 1.06 [99.4% CI, 1.03-1.09]; *P* < .001), non-Hispanic Black race and ethnicity (vs non-Hispanic White: aOR, 1.17 [99.4% CI, 1.13-1.20]; *P* < .001), and each increasing decile of social deprivation index on a scale from 1 (lowest area deprivation) to 10 (highest area deprivation) (aOR, 1.02 [99.4% CI, 1.02-1.03]; *P* < .001) were all significantly associated with longer intervals between hemodialysis and surgery (Figure).^4^

**Table.** Selected Patient, Procedure, and Facility Characteristics by Interval Between Last Preoperative Hemodialysis and Procedure

Characteristic	Procedures, by interval between last hemodialysis and procedure, No. (%)		
	Short (0 or 1 d) (n = 917 635)	Long (2 or 3 d) (n = 203 128)	
Patient characteristics			
Age, y			
Median (IQR)	65.0 (56.0-73.0)		64.0 (55.0-73.0)
18-39	35 127 (3.8)		9117 (4.5)
40-59	285 059 (31.1)		63 505 (31.3)
60-79	485 678 (52.9)		106 545 (52.5)
≥80	111 771 (12.2)		23 961 (11.8)
Sex			
Male	525 174 (57.2)		113 204 (55.7)
Female	392 461 (42.8)		89 924 (44.3)
Race and ethnicity			
Hispanic	177 091 (19.3)		37 154 (18.3)
Non-Hispanic Asian	30 527 (3.3)		6530 (3.2)
Non-Hispanic Black	302 372 (33.0)		75 525 (37.2)
Non-Hispanic White	407 567 (44.4)		83 900 (41.3)
Missing	78 (0.0)		19 (0.0)
Social deprivation index, median (IQR)^a^	7 (5-9)		7 (5-9)
Cause of ESKD			
Diabetes	547 236 (59.6)		114 554 (56.4)
Hypertension	222 943 (24.3)		53 528 (26.4)
Glomerulonephritis	55 725 (6.1)		13 401 (6.6)
Cystic kidney	14 295 (1.6)		3520 (1.7)
Other urologic	10 417 (1.1)		2511 (1.2)
Other or unknown	65 776 (7.2)		15 289 (7.5)
Missing	1243 (0.1)		325 (0.2)
Vascular access type			
Catheter	273 886 (29.8)		64 325 (31.7)
Graft	<147 800 (<16.1)^b^		38 463 (18.9)
Fistula	496 003 (54.1)		97 599 (48.0)
Missing	<10 (<0.0)^b^		<2800 (<1.3)^b^
Rural-urban commuting area code, median (IQR)^c^	1 (1-1)		1 (1-1)
Dialysis schedule			
MWF	624 504 (68.1)		76 431 (37.6)
TThS	293 131 (31.9)		126 697 (62.4)
Listed for kidney transplant	38 989 (4.2)		10 379 (5.1)
Years receiving hemodialysis at time of procedure, median (IQR)^d^	3.3 (1.6-5.7)		3.2 (1.5-5.7)
Prior procedure within 30 d	217 812 (23.7)		36 497 (18.0)
Medicare/Medicaid dual eligible	419 815 (45.7)		98 342 (48.4)
Charlson comorbidities^e^			
Myocardial infarction	262 705 (28.6)		58 727 (28.9)
Congestive heart failure	505 925 (55.1)		112 856 (55.6)
Peripheral vascular disease	512 567 (55.9)		113 750 (56.0)
Cerebrovascular disease	369 252 (40.2)		82 363 (40.5)
Dementia	64 549 (7.0)		15 369 (7.6)
Chronic pulmonary disease	392 175 (42.7)		88 664 (43.6)
Connective tissue disease–rheumatic disease	63 627 (6.9)		14 348 (7.1)
Peptic ulcer disease	90 774 (9.9)		20 317 (10.0)
Mild liver disease	245 349 (26.7)		56 136 (27.6)
Diabetes without complications	571 328 (62.3)		125 986 (62.0)
Diabetes with complications	539 917 (58.8)		118 152 (58.2)
Paraplegia and hemiplegia	61 346 (6.7)		13 716 (6.8)
Cancer	138 629 (15.1)		31 151 (15.3)
Moderate or severe liver disease	31 109 (3.4)		7247 (3.6)
Metastatic carcinoma	24 958 (2.7)		5727 (2.8)
AIDS/HIV	15 301 (1.7)		3765 (1.9)
Charlson comorbidity index, median (IQR)	8 (2-10)		8.0 (2-10)
Procedure characteristics			
RVU of the surgical procedure, median (IQR)	6.3 (1.8-12.0)		9.0 (4.2-13.3)
Procedure day of the week			
Monday	49 832 (5.4)		124 077 (61.1)
Tuesday	280 506 (30.6)		9940 (4.9)
Wednesday	186 981 (20.4)		25 938 (12.8)
Thursday	261 431 (28.5)		18 666 (9.2)
Friday	138 885 (15.1)		24 507 (12.1)
Procedure organ system			
Cardiovascular	298 909 (32.6)		88 102 (43.4)
ENT and dental	2184 (0.2)		492 (0.2)
Endocrine	6184 (0.7)		1605 (0.8)
Eye	351 537 (38.3)		61 814 (30.4)
Gastrointestinal and abdominal	33 862 (3.7)		8545 (4.2)
Hematologic	709 (0.1)		188 (0.1)
Nervous system	7198 (0.8)		1714 (0.8)
Obstetric and gynecologic	1444 (0.2)		457 (0.2)
Orthopedic	38 204 (4.2)		10 200 (5.0)
Skin and breast	159 423 (17.4)		21 487 (10.6)
Thoracic	1199 (0.1)		396 (0.2)
Urologic	16 782 (1.8)		8128 (4.0)
Facility characteristics			
Procedure facility type			
Office	340 422 (37.1)		53 803 (26.5)
Home	2969 (0.3)		325 (0.2)
Inpatient hospital	69 943 (7.6)		29 990 (14.8)
Outpatient hospital	387 167 (42.2)		95 769 (47.1)
Ambulatory surgery center	87 975 (9.6)		19 567 (9.6)
Skilled nursing facility	22 030 (2.4)		2675 (1.3)
Other	7129 (0.8)		999 (0.5)
For-profit facility^f^	801 759 (87.4)		177 676 (87.5)

Abbreviations: ENT, ear, nose, and throat; ESKD, end-stage kidney disease; MWF, Monday-Wednesday-Friday; RVU, relative value unit; TThSat, Tuesday-Thursday-Saturday.

^a^ Social deprivation index was quantified using deciles (integers from 1 to 10), with increasing values representing higher area level deprivation based on seven demographic characteristics collected in the American Community Survey.^4^ Data were available for 905 398 procedures with a short interval and 199 861 with a long interval.

^b^ Exact cell count modified to preserve patient privacy.

^c^ Data available for 915 824 procedures with a short interval and 202 708 with a long interval.

^d^ Data available for less than 940 200 procedures with a short interval and less than 203 140 with a long interval. Exact counts were modified to preserve patient privacy.

^e^ Charlson comorbidities were determined using published coding algorithms.^6^ Kidney disease was not included, as the study cohort only consisted of patients with ESKD. For calculation of the Charlson Comorbidity Index, all patients were considered to have kidney disease.

^f^ Data were available for 909 611 procedures with a short interval and 201 007 with a long interval.

**Figure.**
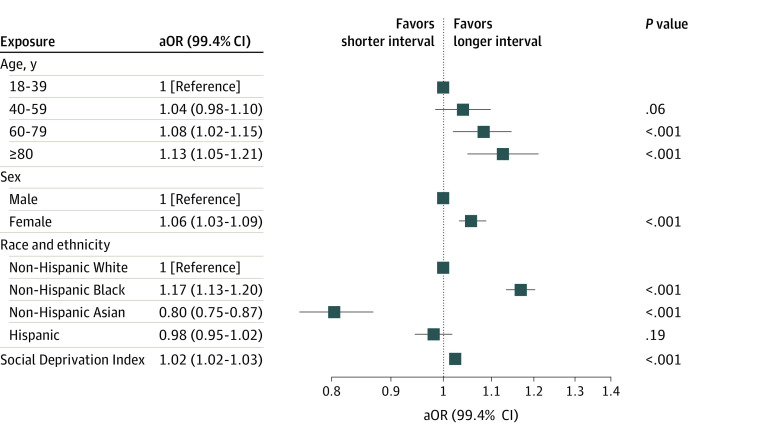
Association of Age, Sex, Race and Ethnicity, and Social Deprivation With the Interval Between Preoperative Hemodialysis and Surgical Procedure Adjusted model estimates reflect Bonferroni-corrected thresholds for statistical significance. Thus, 99.4% CIs are displayed, and *P* < .006 was used to determine statistical significance. Social deprivation index was quantified using deciles (integers from 1 to 10), with increasing values representing higher area level deprivation based on 7 demographic characteristics collected in the American Community Survey.^4^ aOR indicates adjusted odds ratio.

## Discussion

This study found significant age-, sex-, race and ethnicity–, and social deprivation–related disparities in the timing of preoperative hemodialysis among patients with ESKD. Given that a longer interval between preoperative hemodialysis and surgical procedures is associated with higher postoperative mortality, these findings are concerning and identify a possible avenue to improve equity in surgical outcomes for patients with ESKD. Study limitations include residual confounding and limited generalizability to persons who identify as American Indian or Alaska Native, Pacific Islander, Middle Eastern or Arabian or Indian (subcontinent), or those without fee-for-service Medicare. Our study highlights the need for equitable access to perioperative care coordination for persons with ESKD.

## Notes

Supplement 1. **eAppendix.** Supplemental Methods **eTable.** Missing Data for Exposures and Selected Covariates
eReferences.

Supplement 2.
Data Sharing Statement
