# Supplementary material for: Disparities in the Timing of Preoperative Hemodialysis Among Patients With End-Stage Kidney Disease
Source: JAMA Netw Open. 2023 Jul 28;6(7):e2326326. doi: 10.1001/jamanetworkopen.2023.26326 (PMC10383006; doi:10.1001/jamanetworkopen.2023.26326)
Supplement: Supplement 1. — eAppendix. Supplemental Methods eTable. Missing Data for Exposures and Selected Covariates eReferences. [file jamanetwopen-e2326326-s001.pdf]

Supplemental Online Content

Fielding-Singh V, Vanneman MW, Morris AM, Chertow GM, Lin E. Disparities in the timing of preoperative hemodialysis among patients with end-stage kidney disease. *JAMA Netw Open.* 2023;6(7):e2326326. doi:10.1001/jamanetworkopen.2023.26326

eAppendix. Supplemental Methods

eTable. Missing Data for Exposures and Selected Covariates

eReferences.

This supplemental material has been provided by the authors to give readers additional information about their work.

© 2023 Fielding-Singh V et al. *JAMA Network Open.*

eAppendix. Supplemental Methods

Data Source

The United States Renal Data System is a national registry of all patients receiving dialysis for ESKD in the United States irrespective of insurance.[1] The USRDS is linked to Medicare Parts A and B claims for patients insured through fee-for-service (FFS) Medicare.

Covariates and Exposures

In addition to the primary exposures, the model adjusted for day of the week of the procedure, dialysis schedule, cause of ESKD, vascular access, procedure Relative Value Units, procedure facility type, rural-urban commuting area codes,[2] active listing for kidney transplant at the time of procedure, prior surgical procedure within 30 days, dual eligibility for Medicare/Medicaid, facility profit orientation, Charlson comorbidities,[3] and procedure organ system, as previously described.[4]

The patient zip code (from the dialysis facility for the week or, if this was missing, from the Medical Evidence Report Form) was used to estimate social deprivation using the social deprivation index,[5] an estimation of area level deprivation based on seven demographic characteristics collected in the American Community Survey. Social deprivation index was quantified using deciles (integers from 1 to 10), with increasing values representing higher area level deprivation. Patient zip code was cross referenced with 2010 Rural-Urban Commuting Area Codes, a system where whole numbers (1-10) delineate metropolitan, micropolitan, small town, and rural commuting areas based on the size and direction of the primary (largest) commuting flows.[2] The Quan modification of the Charlson comorbidities and comorbidity index were determined for all inpatient, outpatient, and physician/supplier claims that occurred prior to the surgical procedure to identify patients' comorbidities at the time of the procedure.[3]

Model Specifications

For our primary analysis, we estimated a logistic regression model for a longer interval between the most recent preoperative hemodialysis treatment and the surgical procedure. In addition to the covariates above, the model utilized an interaction term between the procedure day of the week and the patient's baseline hemodialysis schedule (Monday-Wednesday-Friday or Tuesday-Thursday-Saturday).

Approach to Missing Data

Observations with missing primary exposure data (age, gender, race and ethnicity, and social deprivation) were not included in the analysis. For all remaining missing data, we imputed five datasets by chained equations using all covariates and the primary outcome.[6] Results were combined using Rubin's standard rules.[7] Missing data are described in the Table and eTable 1.

© 2023 Fielding-Singh V et al. *JAMA Network Open.*

eTable. Missing Data for Exposures and Selected Covariates

Variable	n (%)[a]
Age[b]	0 (0%)
Gender[b]	0 (0%)
Race and Ethnicity[b]	97 (0%)
Social Deprivation Index[b]	15,504 (1.4%)
Cause of ESKD[c]	1,568 (0.1%)
Vascular access type[c]	2,743 (0.2%)
Rural-Urban Commuting Area Code[c]	2,231 (0.2%)
Years on hemodialysis at time of procedure[c]	<20 (0%)[d]
For-profit facility[c]	10,145 (0.9%)

[a] N = 1,120,763.
[b] Observations with missing exposure data were dropped from multivariable models, as described in eAppendix 1.
[c] Missing covariate data were imputed using multiple imputation with chained equations, as described in eAppendix 1.
[d] Exact cell count modified to preserve patient privacy.
Abbreviations: ESKD, end-stage kidney disease.

© 2023 Fielding-Singh V et al. *JAMA Network Open.*

eReferences

1. United States Renal Data System. *2022 USRDS Annual Data Report: Epidemiology of kidney disease in the United States*. 2022. Accessed March 20, 2023. https://adr.usrds.org/2022
2. Robert Graham Center - Policy Studies in Family Medicine & Primary Care. Social deprivation index (SDI). November 5, 2018. Accessed February 28, 2023. https://www.graham-center.org/rgc/maps-data-tools/sdi/social-deprivation-index.html
3. United States Department of Agriculture ERS. Rural-Urban Commuting Area Codes. Accessed January 21, 2023, https://www.ers.usda.gov/data-products/rural-urban-commuting-area-codes/
4. Quan H, Sundararajan V, Halfon P, et al. Coding algorithms for defining comorbidities in ICD-9-CM and ICD-10 administrative data. *Med Care*. Nov 2005;43(11):1130-9.
5. Fielding-Singh V, Vanneman MW, Grogan T, et al. Association Between Preoperative Hemodialysis Timing and Postoperative Mortality in Patients With End-stage Kidney Disease. *JAMA*. Nov 8 2022;328(18):1837-1848. doi:10.1001/jama.2022.19626
6. Sterne JAC, White IR, Carlin JB, et al. Multiple imputation for missing data in epidemiological and clinical research: potential and pitfalls. *BMJ*. 2009;338:b2393. doi:10.1136/bmj.b2393
7. Rubin DB. *Multiple Imputation for Nonresponse in Surveys*. Wiley; 1987.
