# Supplementary material for: Disparities in the Timing of Preoperative Hemodialysis Among Patients With End-Stage Kidney Disease
Source: JAMA Netw Open. 2023 Jul 28;6(7):e2326326. doi: 10.1001/jamanetworkopen.2023.26326 (PMC10383006; doi:10.1001/jamanetworkopen.2023.26326)
Supplement: Supplement 2. — Data Sharing Statement [file jamanetwopen-e2326326-s002.pdf]

Data Sharing Statement

Fielding-Singh. Disparities in the Timing of Preoperative Hemodialysis Among Patients With End-Stage Kidney Disease. *JAMA Netw Open*. Published July 28, 2023. doi:10.1001/jamanetworkopen.2023.26326

Data
Data available: No

Additional Information
Explanation for why data not available: The USRDS Coordinating Center provides data to anyone who submits a qualified request and receives approval by the NIH-NIDDK. However, per our data use agreement with the USRDS we are unable to share the data directly.
